# Modulating rice starch physicochemical and *in vitro* digestive properties with ball-milled pea protein isolate: Effect of ball-milling time

**DOI:** 10.1016/j.fochx.2026.104466

**Published:** 2026-09-19

**Authors:** Jun Jiang, Yirong Zhang, Jun Wang, Derui Kong, Tianyu Yang, Zuohua Zhu, Naifu Wang

**Affiliations:** a Key Laboratory of Jianghuai Agricultural Product Fine Processing and Resource Utilization of Ministry of Agriculture and Rural Affairs, College of Food and Nutrition, Anhui Agricultural University, Hefei 230036, China; b School of Cultural Tourism and Education, Anhui Technical College of Industry and Economy, Hefei 230036, China; c Institute of Bast Fiber Crops & Center of Southern Economic Crops, Chinese Academy of Agricultural Sciences, Changsha 410205, China

**Keywords:** Particle size reduction, Starch-protein interaction, Starch swelling, *In vitro* digestibility

## Abstract

Strengthening starch–protein intermolecular interactions is critical for modulating the physicochemical properties of starch *via* proteins. In this study, pea protein isolate (PPI) was pretreated by ball-milling for different durations (0, 10, 20, 30, 40 min), and the effects of milled PPI on the physicochemical properties and *in vitro* digestibility of rice starch (RS) were investigated. Compared with native PPI, ball-milling treatment of PPI (40 min) reduced the swelling power and water absorption capacity of PPI-RS mixtures by 5.38% and 6.39%, respectively, while increasing their solubility by 21.25%. Furthermore, ball-milling treatment of PPI weakened the short-range order of starch molecules and decreased the gelatinization enthalpy, peak viscosity, trough viscosity, final viscosity, breakdown, setback, gel hardness of PPI-RS composite mixtures. *In vitro* digestion tests revealed that ball-milling treatment of PPI (40 min) retarded starch digestion in PPI-RS composites, resulting in a 23.78% increase in resistant starch content. Notably, no significant further changes (*p* > 0.05) in the values of gelatinization enthalpy, setback value, gel hardness, and rapidly digestible starch content were detected when the ball-milling duration exceeded 20 min. This work can provide useful guidelines for developing novel starch-based foods with high nutritional quality and low glycemic index.

## 1 Introduction

Starch is a major component of numerous foods and strongly influences the texture and consumer acceptability of starch-based products. Besides serving as a crucial energy source for human nutrition, starch is also widely used in the food industry as a thickener, stabilizer, gelling agent, and water retention agent. However, native starch exhibits some inherent drawbacks, such as low solubility, poor shear and thermal resistance, high retrogradation propensity, and rapid digestibility, which greatly limit its industrial applicability (Chen et al., 2019; Yu et al., 2020). To meet the industrial requirements and expand starch applications in food industry, incorporating dietary compounds into starch matrices is a promising route to synchronously enhance the nutritional quality and functional performance of starch (Song et al., 2026). Among various dietary components, plant proteins have gained more research attention, owing to their essential nutritional value and the growing market popularity of plant-based protein products. For example, Liu et al. (2026) found that soy protein incorporation disrupted the intermolecular interactions of wheat starch chains and reduced starch gel viscosity. Zhang et al. (2026) reported that buckwheat protein hydrolysate incorporation promoted the ordered crystalline arrangement of starch, and reduced the viscosity of buckwheat starch pastes. According to the study of Liu and Ouyang (2025), proteins can adhere to the surface of starch granules to form a physical barrier and interact with starch molecules *via* multiple intermolecular forces (Fig. 1), consequently modulating the physicochemical properties of starch.

**Fig. 1:**
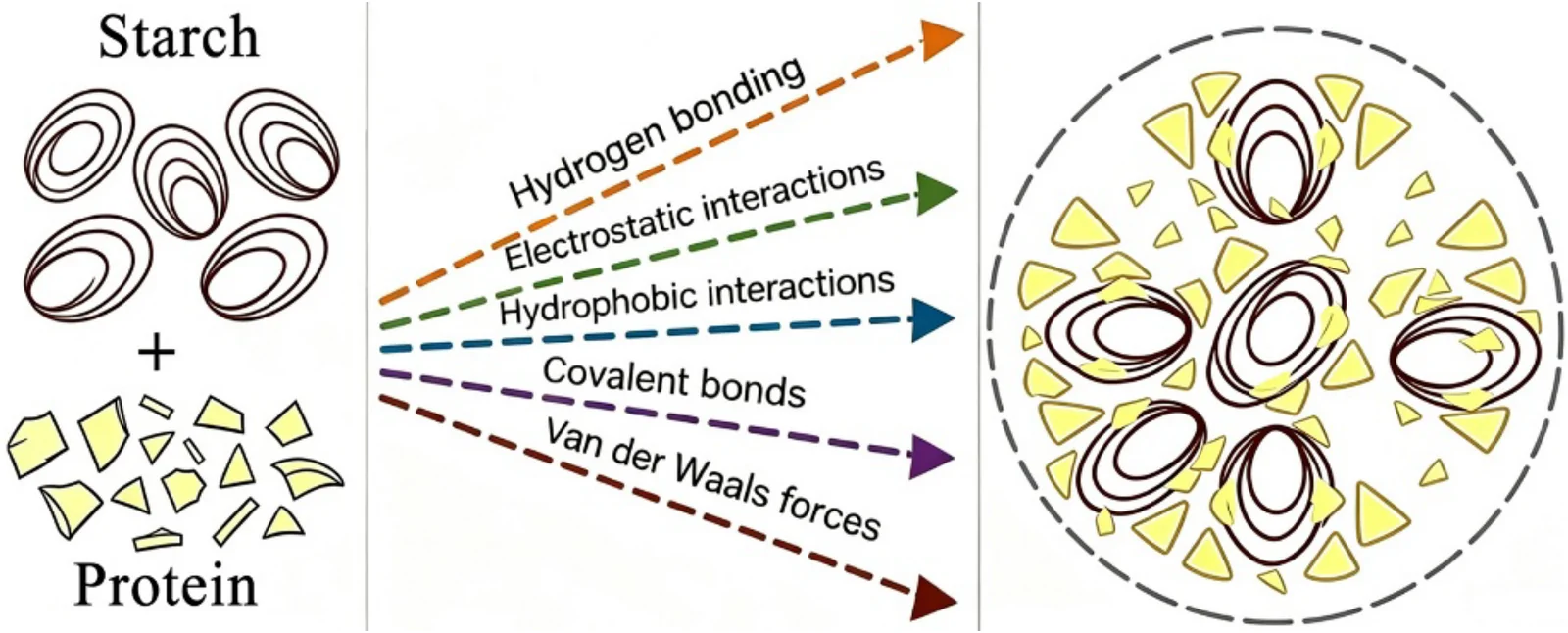
Overview of the intermolecular forces between protein–starch.

The key to regulate the physicochemical properties of starch using proteins is how to strengthen the interactions between starch and proteins (Li, He, et al., 2024). Cheng et al. (2025) found zein nanoparticles exhibited high compatibility with the pea starch-soy protein isolate matrix, thus enhancing the structural properties of pea starch-soy protein isolate films by forming hydrogen bonds between the nanoparticles and the biopolymer network. As a cost-effective and energy-efficient physical modification technology, ball-milling has gained extensive attention for modulating the functional properties of proteins in recent years (Liu et al., 2024). This technique can disrupt the intrinsic molecular interactions of proteins, such as disulfide bonds and hydrophobic interactions, reduce particle size, increase surface area and expose more functional groups, thereby effectively improving the solubility, emulsifying ability, and water holding capacity of proteins (Li, Li, et al., 2024; Zhang et al., 2025). When the proteins with reduced particle size after ball-milling treatment are incorporated into starch matrices, their increased surface activity might strengthen the starch-protein interactions, providing a feasible approach to regulate the physicochemical properties of starch.

To date, most existing studies have primarily focused on the effects of protein varieties or hydrolyzed protein fractions on the physicochemical properties of starch. Nevertheless, limited research is available regarding the modulation of starch physicochemical properties by ball-milled proteins. The main component in rice is starch (about 90% of rice dry weight), which largely determines the quality of rice-based products, such as rice noodles, rice cake, and rice ball. In order to improve the physicochemical properties of rice starch (RS), this study first modified pea protein isolate (PPI) *via* ball-milling with varied durations, and further investigated the effects of ball-milled PPI on the gelatinization, pasting, and retrogradation behaviors as well as the *in vitro* digestibility of RS. The findings will deepen the current understanding of how modified proteins regulate starch physicochemical properties and provide useful guidance for developing novel starch-based products with high nutrition quality and low-glycemic index.

## 2 Materials and methods

### 2.1 Materials

Pea protein isolate (PPI, 89.5% protein, w/w) was bought from Junta Co., Ltd. (Xian, China). Rice starch (RS, 95.4% starch, amylose 17.8%) was purchased from Shenhua Co., Ltd. (Dalian, China). Porcine pancreatic α-amylase (100 U/mg) and amyl glucosidase (70 U/mg) were bought from Sigma-Aldrich, Inc. (St. Louis, MO, USA).

### 2.2 Preparation of modified PPI by ball-milling

A planetary ball mill (YD-YXQM, Misi Instrument Co., Ltd., Changsha, China) was used for the PPI modification and the ball-milling process was performed according to the method of Liu, Zheng, et al. (2021). PPI sample (20 g) of was poured into a 500 mL ball-milling jar containing 100 small (diameter 6 mm), 50 medium (diameter 10 mm), and 5 large (diameter 20 mm) stainless steel grinding beads. The rotation rate was set at 500 r/min, with the rotation direction alternating every 5 min to ensure uniform grinding. Liquid nitrogen was continuously supplied during processing to maintain the temperature between −20 °C and −10 °C. Samples were collected at intervals of 10, 20, 30, and 40 min of milling and labeled as PPI10, PPI20, PPI30, and PPI40, respectively. Native PPI was labeled as the PPI0.

### 2.3 Determination of morphology, particle size distribution, zeta potential, and surface hydrophobicity of PPI

The morphology of PPI was observed by a scanning electron microscope (SEM, S-4800, Hitachi Ltd., Tokyo, Japan) at an accelerating voltage of 3.0 kV. PPI powder was evenly distributed onto double-sided adhesive tape and sputter-coated with gold for 30 s. A magnification of 500 × was selected to observe the surface morphology of PPI. The particle size distribution of PPI was determined using a laser diffraction particle size analyzer (Mastersizer 2000, Malvern Panalytical Ltd., Worcester, UK). A quantity of 5.0 g of PPI was added to 50 mL of phosphate buffer (pH 7.0, 0.01 M). The mixture was mixed thoroughly for 1 h at room temperature using a magnetic stirrer (HJ-3, Jincheng Experimental Ltd., Jiangsu, China) at 500 r/min to ensure complete homogenization. Then the PPI dispersion was poured into the sample cell of the particle size analyzer and mixed at 1500 r/min. Measurement commenced when the obscuration reached 10%–15%.

The zeta potential of PPI was measured by the laser particle size analyzer mentioned above according to the method of Li, He, et al. (2024). The PPI solution was prepared at a concentration of 1 mg/mL in 10 mM PBS solution (pH 7.0) and filtered through a 0.45 μm Millipore membrane. The surface hydrophobicity of PPI was determined following the method described by Li, Li, et al. (2024). An appropriate amount of PPI was dissolved in 10 mM PBS solution (pH 7.0), and the resulting PPI solution was diluted using PBS to obtain gradient concentrations of 0.02, 0.04, 0.06, 0.08, and 0.1 mg/mL. Then, 4 mL of the above diluted solution was mixed with 20 μL of 8 mM 8-anilino-1-naphthalenesulfonate (ANS) solution (prepared in PBS). The mixed samples were incubated in the dark for 10 min prior to fluorescence detection. Fluorescence intensity was measured using a fluorescence spectrometer (F-2710, Hitachi Ltd., Tokyo, Japan) with the following parameters: excitation wavelength of 390 nm, emission wavelength of 470 nm, and a slit width of 5 nm. The fluorescence intensity was plotted against protein concentration, and the slope of the initial linear portion of the fitted curve was taken as the surface hydrophobicity.

### 2.4 Preparation of PPI-RS mixtures

PPI-RS mixtures were prepared according to the method of Kadival et al. (2023) with some modifications. RS was dispersed in distilled water to form a 5 g/100 g (w/w) starch slurry. PPI powders with different ball-milling durations were subsequently incorporated into the starch slurry at a level of 10% (w/w, based on dry starch weight). The mixtures were stirred using a magnetic stirrer (HJ-3, Jincheng Experimental Ltd., Jiangsu, China) at room temperature for 1 h. After freeze-drying, the obtained PPI-RS mixtures were labeled as PPI0-RS, PPI10-RS, PPI20-RS, PPI30-RS, and PPI40-RS, respectively. Pure RS without PPI addition was set as the control group.

### 2.5 Fourier transform infrared spectroscopy (FTIR) analysis

Pure RS and PPI-RS mixture samples were mixed with KBr powder at a 1:100 (w/w) ratio in an agate mortar and pressed into round tablets. The prepared tablets were scanned using a FTIR spectrometer (Nicolet 6700, Thermo Electric Corporation, Waltham, MA, USA) in the range of 4000–400 cm^−1^ with a resolution of 2 cm^−1^. The spectral region of 1200–800 cm^−1^ was deconvoluted using OMNIC 8.0 software, and the peak absorbance ratios of 995 cm^−1^/1022 cm^−1^ and 1047 cm^−1^/1022 cm^−1^ were calculated to evaluate the short-range molecular order of starch (Chen et al., 2019).

### 2.6 Solubility, swelling power, and water absorption capacity

The solubility, swelling power, and water absorption capacity of the pure RS and PPI-RS mixtures were determined according to the method of Sun and Xiong (2014) with minor modifications. An amount (1 g) of RS or PPI-RS mixture was dispersed in 40 mL of distilled water using a 100 mL centrifuge tube. The suspension was continuously stirred using a magnetic stirrer in a water bath at 90 °C for 30 min, then centrifuged at 4000 r/min for 15 min. Then the supernatant was decanted and the swollen sediment was weighed. A 10 mL aliquot of the supernatant was transferred to a Petri dish and dried in an air oven at 120 °C to a constant mass. Solubility, swelling power and water absorption capacity were calculated using the following equations:

$$\text{Solubility}\;\left(\%\right)=\frac{\text{Weight of dried supernatan}\;t\times 4}{\text{Weight of sample}}\times 100\%$$

$$\text{Swelling Power}=\frac{\text{Weight of swollen particles}}{\text{Weight of sample}\times \left(1-\text{Solubility}\right)}$$

$$\text{Water absorption capacity}=\frac{\text{Weight of swollen particles}}{\text{Weight of sample}}$$

### 2.7 Thermal properties

The thermal properties of PPI-RS mixtures were investigated using a DSC8000 differential scanning calorimeter (DSC, PerkinElmer Inc., Waltham, MA, USA). A sample suspension with a mass fraction of 30% was prepared using distilled water and equilibrated for 24 h at 25 °C. Subsequently, 800 mg of the sample suspension and distilled water (reference) were accurately weighed into the sample pan and reference pan, respectively. The samples were heated from 30 °C to 90 °C at a rate of 1 °C/min. The onset temperature (To), peak temperature (Tp), conclusion temperature (Tc), and gelatinization enthalpy (Δ*H*) associated with the thermal transitions were determined.

### 2.8 Pasting properties

The pasting properties of PPI-RS mixtures were analyzed using a Super 3 rapid visco analyzer (RVA, Newport Scientific Pty Ltd., Warriewood, Australia). A quantity of 3.5 g (14% w/w) of the PPI-RS mixture was weighed into the aluminum canister, and then added 25 mL of distilled water. The RVA measurement was carried out using the following program: The slurry was held at 50 °C for 1 min, heated from 50 °C to 95 °C in 4 min, held at 95 °C for 2.5 min, cooled to 50 °C in 4 min, and held at 50 °C for 1.5 min. The pasting parameters such as peak viscosity (PV), trough viscosity (TV) breakdown (BD), final viscosity (FV), and setback (SB) were determined.

### 2.9 Texture profile analysis (TPA)

TPA was performed according to the method of Sun and Xiong (2014). After the RVA test, the PPI-RS mixtures in the RVA canisters were cooled, sealed, and stored for 24 h at 4 °C to form gels. The TPA of the gels was performed using a TA. XT Plus texture analyzer (Stable Micro Systems, Godalming, UK) equipped with a P/0.5 probe. The pre-test, test, and post-test speeds were all 1.0 mm/s. The compression strain was 50%, and the trigger force was 5 g.

### 2.10 Determination of digestibility

The digestibility of starch was determined according to the method described by Wu et al. (2021) with minor modifications. Briefly, a 400 mg sample of the PPI-RS mixture was dispersed in 15 mL of sodium acetate buffer (0.2 M, pH 5.2, containing 1.0 mM CaCl₂) in a 100-mL Erlenmeyer flask containing six glass beads (5 mm diameter). After incubating the suspension in a shaking water bath at 37 °C for 10 min (160 r/min), 3 mL of a mixed enzyme solution (α-amylase 100 U/mL and glucoamylase 100 U/mL) was added to initiate an enzymatic hydrolysis. During hydrolysis, 0.5 mL of hydrolysate was collected at 0, 20, and 120 min. The enzymatic reaction was immediately terminated by adding 4.5 mL of absolute ethanol. The resulting mixtures were then centrifuged at 5000 ×*g* for 10 min. The glucose content in the supernatant was determined using a GOPOD method kit (Jiancheng Biochemistry Co., Ltd., China). The contents of rapidly digestible starch (RDS), slowly digestible starch (SDS), and resistant starch were calculated using the following formulas: RDS = [(M_20_ - M_0_) × 0.9]/M_TS_ × 100%, SDS = [(M_120_ − M_20_) × 0.9]/M_TS_ × 100%, resistant starch = (1 − RDS − SDS) × 100%. In the formulas, M_0_, M_20_, M_120_ are the amount of glucose released after 0 min, 20 min and 120 min, respectively. M_TS_ is the total mass of starch.

### 2.11 Statistical analysis

All measurements were performed three times and results were expressed as means ± standard deviation. Statistical analysis was conducted by one-way analysis of variance (ANOVA) using SPSS 25.0. *p* < 0.05 (Duncan's test) was considered significant and Tukey's HSD test was additionally performed to verify the robustness of pairwise comparison results.

## 3 Results and discussion

### 3.1 Morphology, particle size distribution, zeta potential, and surface hydrophobicity of PPI

The morphology of PPI before and after the ball-milling treatment was investigated using SEM at a magnification of 500×. As shown in Fig. 2, native PPI particles displayed intact spherical geometry with smooth surfaces but heterogeneous particle sizes. As the ball-milling time increased, the particle size of PPI decreased markedly. The PPI powder was fragmented, appearing as homogeneous, rough, flaky fragments. When the ball-milling time exceeded 30 min, the original spherical structure of PPI was completely disrupted. The ball-milled PPI, featuring smaller particle size and rougher surface, may possess enhanced interaction potential with starch and consequently alter the physicochemical properties of starch.

**Fig. 2:**
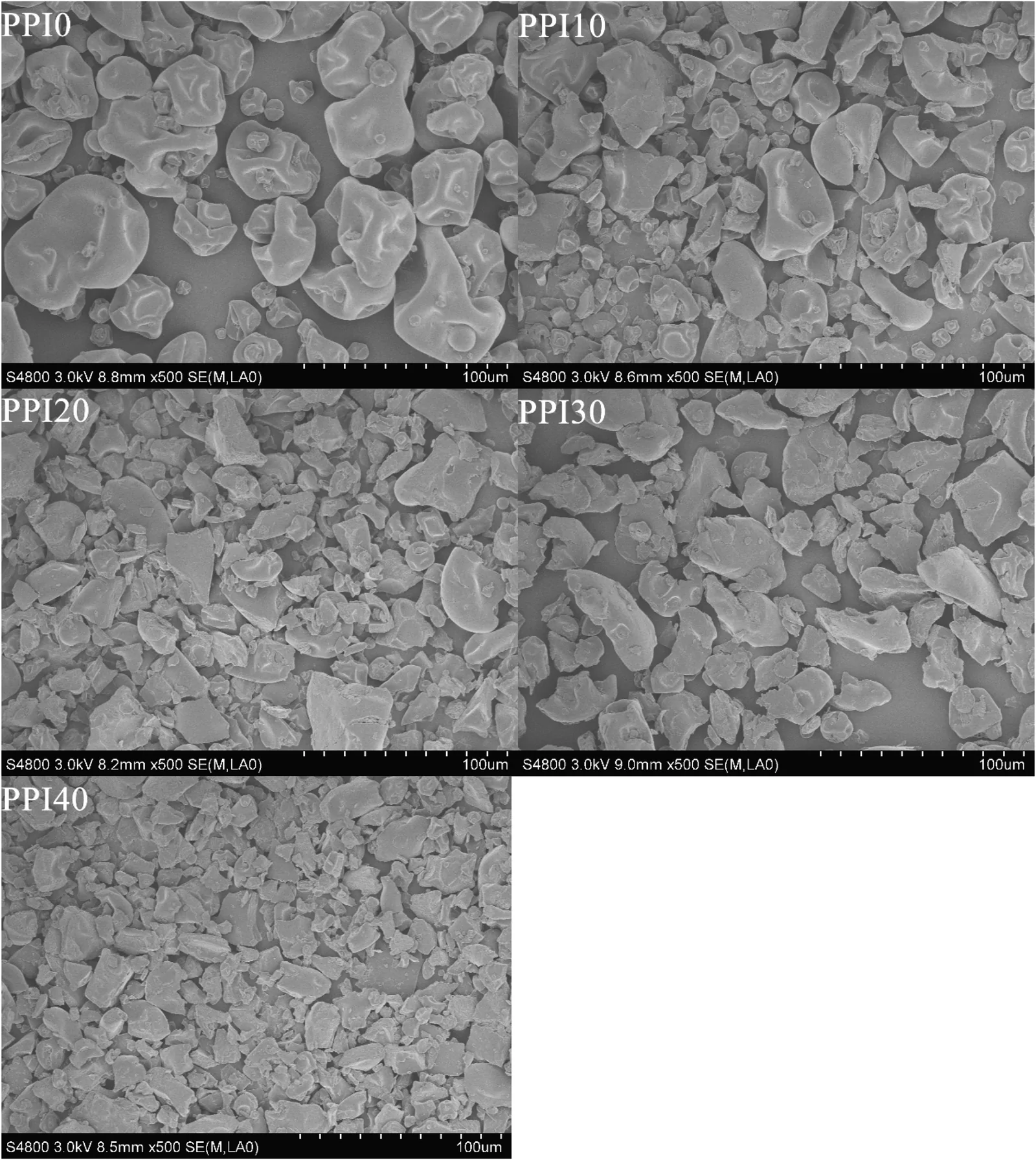
Morphology (500 × magnification, scale bar = 100 μm) of ball-milled pea protein isolate (PPI) at different treatment durations. PPI0, PPI10, PPI20, PPI30, and PPI40 correspond to PPI samples ball-milled for 0, 10, 20, 30, and 40 min, respectively.

The effect of ball-milling on the particle size distribution of PPI is shown in Fig. 3A. With the increase of milling time, the particle size distribution of PPI gradually shifted toward smaller particle sizes. Native PPI (PPI0) exhibited a broad particle size distribution ranging from 0 to 500 μm, whereas the ball-milled samples showed a narrower distribution range. These results indicated that ball-milling not only decreased the particle size of PPI but also increased its uniformity. However, when the milling time exceeded 20 min, the reduction in particle size was less pronounced. Liu, Zheng, et al. (2021) observed a similar phenomenon when the ball-milling time of wheat gluten protein exceeded 20 min. This phenomenon may be attributed to the higher energy input required for further particle size reduction. Moreover, ball-milling decreased the particle size of PPI while increasing its specific surface area, thereby exposing more hydrophobic groups and water-binding sites on the PPI surface and promoting particle agglomeration (Chen et al., 2022; Yu et al., 2018).

**Fig. 3:**
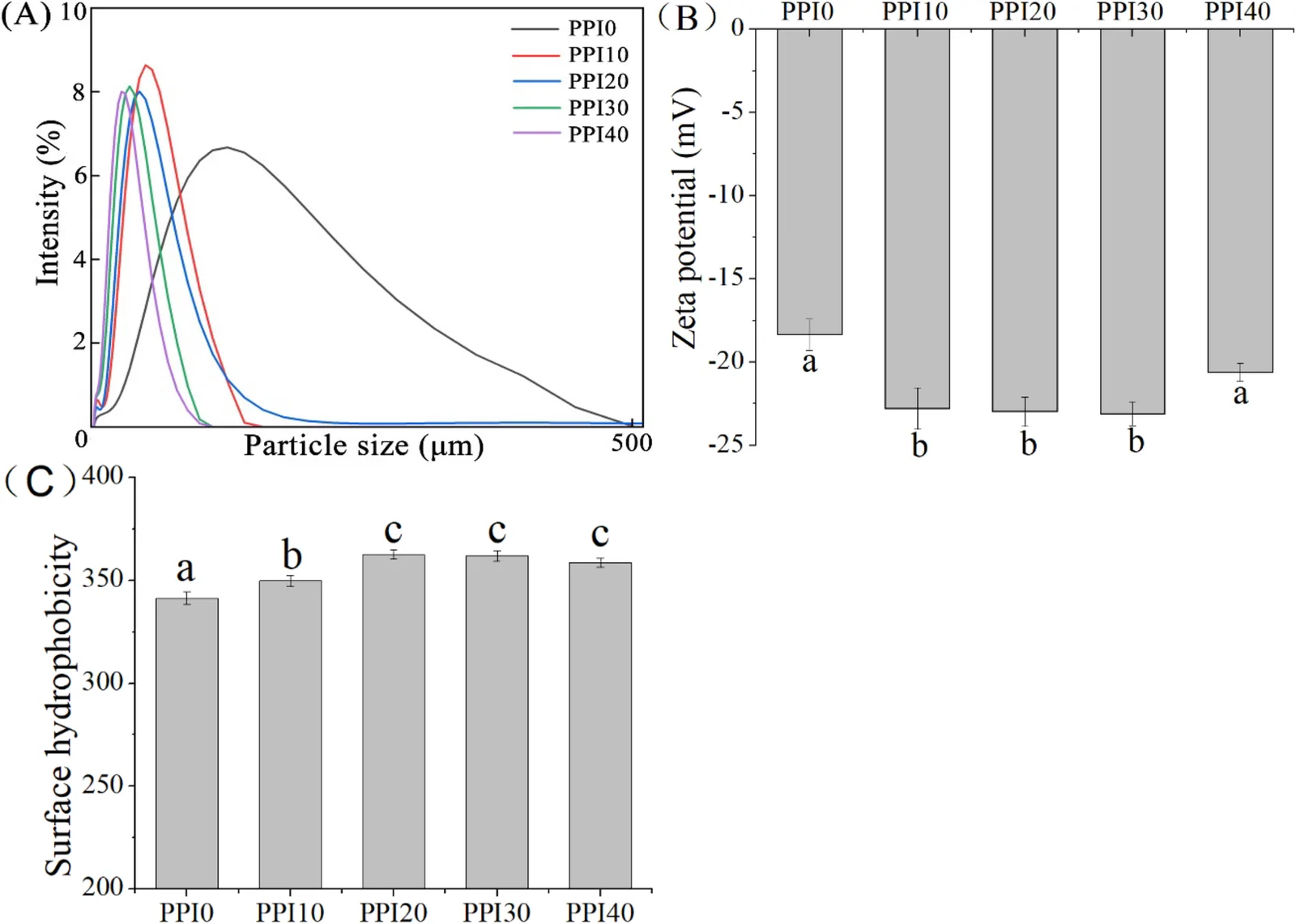
Particle size distribution (A), zeta potential (B), and surface hydrophobicity (C) of ball-milled pea protein isolate (PPI) at different treatment durations. PPI0, PPI10, PPI20, PPI30, and PPI40 correspond to PPI samples ball-milled for 0, 10, 20, 30, and 40 min, respectively.

Zeta potential characterizes the net surface charge of protein molecules, originating either from the cleavage of functional groups or ion adsorption from the continuous phase where proteins are dispersed (Julakanti et al., 2023). Protein particles with high zeta potential can maintain stable dispersions owing to strong electrostatic repulsion, whereas those with low zeta potential tend to aggregate, as attractive intermolecular forces prevail over repulsive forces (Li et al., 2023). As shown in Fig. 3B, native PPI exhibits a zeta potential of −18.35 mV. When the ball-milling time increased from 0 min to 10 min and 20 min, the corresponding zeta potential values rose to −22.78 mV and −22.98 mV, respectively. This finding can be attributed to the particle size reduction and molecular unfolding induced by ball-milling, which exposes a greater number of polar residues on protein surfaces (Wu et al., 2022). However, when the ball-milling time exceeded 20 min, the absolute value of zeta potential of PPI showed a decreasing trend. A similar trend (initial rise followed by subsequent drop in zeta potential) was reported by Gao et al. (2022), who processed commercial PPI using high-intensity ultrasound at 100–400 W. A possible explanation for this phenomenon is that excessive ball-milling exposes internal hydrophobic groups of proteins, strengthens hydrophobic interactions, and forms soluble aggregates, which consequently reduces the net surface charge of PPI.

Surface hydrophobicity quantifies the exposure degree of hydrophobic groups in proteins and can be used to reflect the changes of protein tertiary structure. As shown in Fig. 3C, the surface hydrophobicity of PPI increased significantly (*p* < 0.05) when the ball-milling time increased from 0 min to 10 min and 20 min. This finding agrees with Li, He, et al. (2024), who reported that the surface hydrophobicity of *Cyperus esculentus* protein increased with the increase of ball-milling time. Such behavior originates from mechanical forces (collision, friction and shear) generated during ball- milling, which disrupt the tertiary conformation and aggregation state of PPI, consequently exposing buried hydrophobic groups to the molecular surface (Chen et al., 2022). When the ball-milling time exceeded 20 min, the surface hydrophobicity of PPI exhibited a decreasing trend, but it was still higher than that of native PPI. Two plausible mechanisms may explain this trend: hydrophobic groups reach their maximum surface exposure, or the aggregation of hydrophobic groups buries the binding sites for ANS probes.

### 3.2 FTIR spectroscopy analysis

FTIR spectroscopy is widely used to characterize molecular-level structural variations of starch, including crystallinity, chain conformation and retrogradation (Hu et al., 2015). The diagnostic absorption bands of starch are located within the 800–1200 cm^−1^ region. Therefore, the characteristic peaks in this region were deconvoluted, which are shown in Fig. 4. The absorbance ratio of 995 cm^−1^/1022 cm^−1^ (represents the order degree of starch double helices) and the ratio of 1047 cm^−1^/1022 cm^−1^ (represents the starch crystallinity) can reflect the starch short-range molecular order (Chai et al., 2025). As shown in Table 1, the absorbance ratios of 995 cm^−1^/1022 cm^−1^ and 1047 cm^−1^/1022 cm^−1^ for pure RS were 1.08 and 1.72, respectively. Incorporation of native PPI induced significant reductions (*p* < 0.05) in both ratios of RS, demonstrating a decline in starch short-range molecular order. Lin et al. (2022) also found adding 12% rice protein decreased the short-range molecular order of starch in wheat starch-rice protein complexes. They thought that protein and starch could form a uniform state, which inhibited the interaction between starch molecular chains, thus decreased the starch molecular order.

**Fig. 4:**
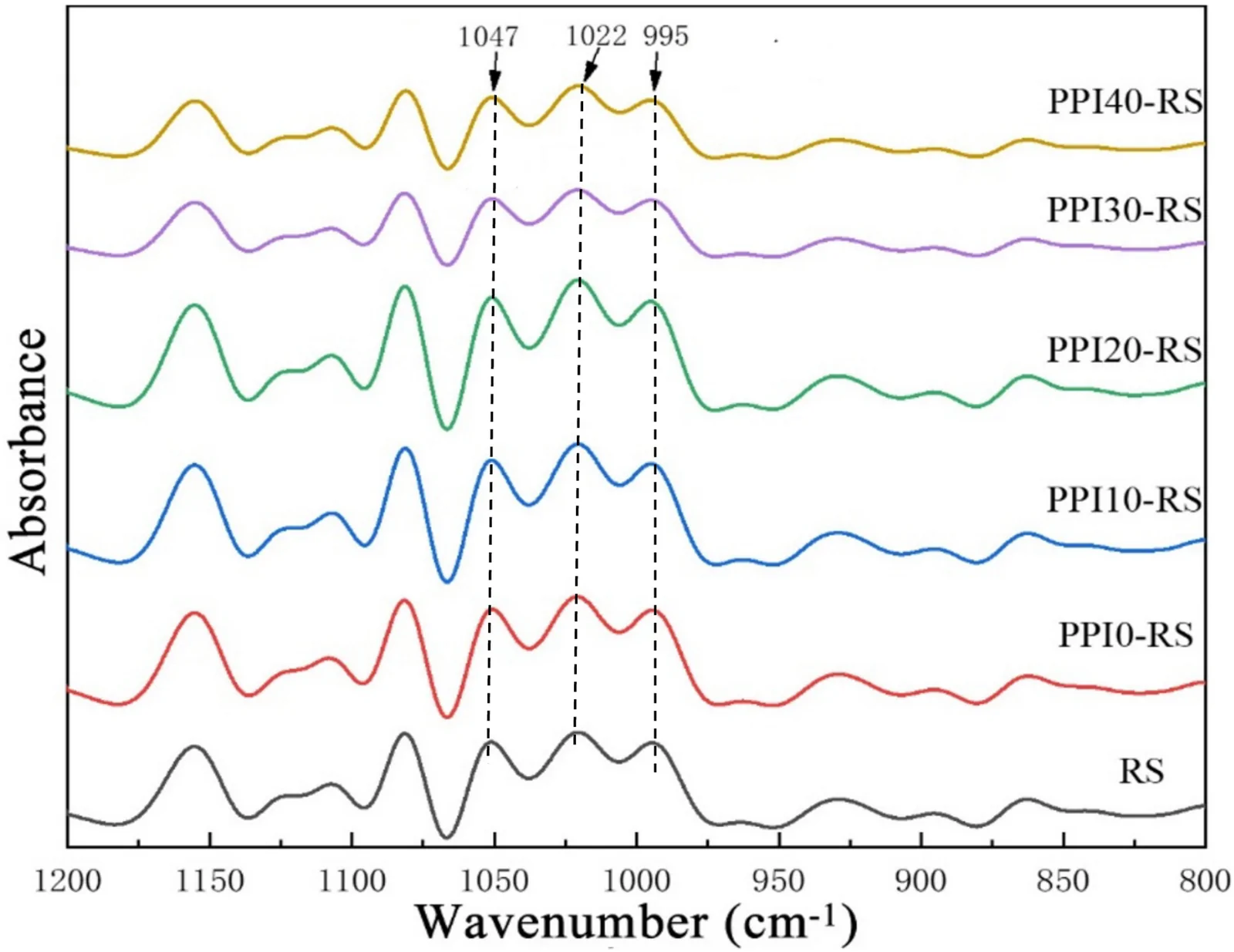
Deconvoluted FTIR spectra of rice starch (RS) and PPI-RS mixtures. RS: rice starch; PPI: pea protein isolate. PPI0-RS, PPI10-RS, PPI20-RS, PPI30-RS, and PPI40-RS denote RS mixed with PPI subjected to ball-milling for 0, 10, 20, 30, and 40 min, respectively.

**Table 1:** Short-range ordered structure parameters of RS or RS in PPI-RS mixtures.

Samples	995 cm^−1^/1022 cm^−1^	1047 cm^−1^/1022 cm^−1^
RS	1.08 ± 0.02^a^	1.72 ± 0.02^a^
PPI0-RS	1.03 ± 0.02^b^	1.65 ± 0.02^b^
PPI10-RS	0.91 ± 0.02^cd^	1.59 ± 0.02^c^
PPI20-RS	0.90 ± 0.01^cd^	1.55 ± 0.02^d^
PPI30-RS	0.93 ± 0.02^c^	1.55 ± 0.01^d^
PPI40-RS	0.88 ± 0.02^d^	1.49 ± 0.15^e^

Values with different letters in the same column are considered significant different (*p* < 0.05). Abbreviations (RS, PPI, PPI0-RS, PPI10-RS, PPI20-RS, PPI30-RS, and PPI40-RS) are the same as those shown in Fig. 4.

Compared with the PPI-RS mixture prepared with native PPI, ball-milling PPI for 0–10 min significantly (*p* < 0.05) decreased the 995 cm^−1^/1022 cm^−1^ and 1047 cm^−1^/1022 cm^−1^ absorbance ratios of starch in the mixtures, indicating that ball-milling further reduced the short-range molecular order of starch. The short-range ordering of starch is mainly maintained by hydrogen bonding (Lu et al., 2016). Proteins can adhere to the starch granule surface through hydrogen bonds, electrostatic complexation, van der Waals forces or hydrophobic interaction, which disrupt the original hydrogen bonds of starch and result in the rearrangement of starch chains (López-Barón et al., 2018; Lu et al., 2016). Ball-milling decreased the particle size of PPI and strengthened the starch-protein interaction, thereby further decreased the starch short-range molecular order in the PPI-RS mixtures. However, when the ball-milling exceeded from 20 to 30 min, the 995 cm^−1^/1022 cm^−1^ ratio increased from 0.90 to 0.93, although the difference was not statistically significant (*p* > 0.05). This increase might be explained by the dual effects of mechanical milling and particle agglomeration. During the early milling stage (≤20 min), continuous mechanical force effectively reduced the particle size of PPI, improved its structural uniformity, and correspondingly decreased the above 995 cm^−1^/1022 cm^−1^ ratio of starch. However, further prolonged milling beyond 20 min provided excessive mechanical energy input, which induced the particle agglomeration phenomenon as mentioned above (as described in Section 3.1). The re-aggregation of fine particles weakened the structural modification effect of ball-milling, thereby reversing the changing trend of the 995 cm^−1^/1022 cm^−1^ ratio of starch. Collectively, these results indicate that moderate ball-milling is required to improve the physicochemical properties of PPI-RS mixture.

### 3.3 Solubility, swelling power, and water absorption capacity

The effects of ball-milled PPI on the solubility, swelling power, and water absorption capacity of RS are presented in Table 2. Swelling power is an important property of gelatinized starch, which is closely related to starch structural integrity, water binding capability, and the strength of intermolecular hydrogen bonds (Liu, Liu, et al., 2021). As shown in Table 2, the addition of PPI significantly (*p* < 0.05) reduced the swelling power of RS. Similar to our results, Liu, Liu, et al. (2021) also observed that whey protein addition significantly reduced the swelling power of potato starch. The reason might be that proteins can bind more water than starch in food systems, competing for water with starch and preventing its full gelatinization (Bühler et al., 2022). Furthermore, proteins can wrap starch granule surfaces and constrain granule swelling (Likitwattanasade & Hongsprabhas, 2010). Compared with native PPI, PPI-RS mixtures containing ball-milled PPI exhibited lower swelling power. Ball-milling disrupted the PPI's spatial structure, and exposed numerous internal hydrophilic groups (as described in Section 3.1: zeta potential). These groups compete with starch for water, consequently decreasing its swelling power. Previous studies have demonstrated that starch swelling power is positively correlated with digestibility (Liu, Yang, et al., 2021). Accordingly, the decreased swelling capacity of starch in PPI-RS mixtures may help reduce its digestibility. The water absorption capacity of starch is positively correlated with its swelling power (Loos et al., 1981). As shown in Table 2, ball-milled PPI with different milling durations altered the water absorption capacity of RS following a trend consistent with that of swelling power.

**Table 2:** The swelling power, water absorption capacity, and solubility of RS or PPI-RS mixtures.

Samples	SP (g/g)	WAC (g/g)	Solubility (%)
RS	7.183 ± 0.006^a^	6.838 ± 0.003^a^	4.80 ± 0.04^e^
PPI0-RS	6.900 ± 0.011^b^	6.564 ± 0.011^b^	4.88 ± 0.02^d^
PPI10-RS	6.807 ± 0.017^c^	6.472 ± 0.015^c^	4.92 ± 0.01^d^
PPI20-RS	6.727 ± 0.005^d^	6.372 ± 0.006^e^	5.27 ± 0.04^c^
PPI30-RS	6.748 ± 0.010^d^	6.374 ± 0.008^e^	5.54 ± 0.05^b^
PPI40-RS	6.796 ± 0.008^c^	6.401 ± 0.006^d^	5.82 ± 0.03^a^

Values with different letters in the same column are considered significantly different (*p* < 0.05). SP, swelling power; WAC, water absorption capacity; Abbreviations (RS, PPI, PPI0-RS, PPI10-RS, PPI20-RS, PPI30-RS, and PPI40-RS) are the same as those shown in Fig. 4.

Compared with pure RS, PPI-RS mixtures exhibited higher solubility. Such solubility enhancement could be attributed to the effect of ionic bonds on PPI surface (as described in Section 3.1: zeta potential). Zhuang et al. (2024) found that cation addition increased the solubility of potato starch, as cations can disrupt starch crystalline domains and promote starch molecule leaching. Sun and Xiong (2014) reported that the solubility of pea starch-peanut protein mixtures increased with the increase of protein ratio, since peanut protein contained many charged amino acids, which could hinder the development of starch crystalline regions. As the ball-milling time increased, the solubility of the PPI-RS mixtures increased accordingly. Ball-milling disrupted the spatial structure of PPI and exposed more charged amino acids, which could increase the surface electric charge and optimize the dispersion behavior of PPI-RS mixtures, consequently resulting in a higher solubility.

### 3.4 Thermal properties

The thermal parameters of the pure RS and PPI-RS mixtures are shown in Table 3. Compared with pure RS, the addition of native PPI significantly (*p* < 0.05) increased the gelatinization temperatures (*T₀*, *T*_*p*_, and *T*_c_) of RS, while significantly reducing (*p* < 0.05) its Δ*H* value. Similar to our results, Yang et al. (2019) also found that whey protein addition significantly increased the gelatinization temperatures (*T₀*, *T*_*p*_, and *T*_c_) and decreased the Δ*H* value of corn starch. Water availability is critical for starch gelatinization and higher water content facilitates starch gelatinization (Yang et al., 2019). The addition of PPI can compete available water with starch in the PPI-RS mixtures and reduce the amount of water entering the starch granules during heating process, thus reducing the starch gelatinization degree and increasing its gelatinization temperatures (Likitwattanasade & Hongsprabhas, 2010; Lin et al., 2022). Furthermore, López-Barón et al. (2017) reported that higher pea protein content enhanced protein–starch interactions in pea protein–wheat starch mixtures, suppressing starch hydration and increasing gelatinization temperature.

**Table 3:** Thermal properties of RS or PPI-RS mixtures.

Samples	T_o_ (°C)	T_p_ (°C)	T_c_ (°C)	Δ*H* (J/g)
RS	58.62 ± 0.02^e^	63.02 ± 0.01^c^	66.96 ± 0.01^e^	11.18 ± 0.04^a^
PPI0-RS	59.70 ± 0.02^d^	63.11 ± 0.02^b^	67.72 ± 0.02^a^	8.89 ± 0.01^b^
PPI10-RS	60.03 ± 0.02^a^	63.16 ± 0.03^b^	67.54 ± 0.03^b^	8.22 ± 0.02^c^
PPI20-RS	59.92 ± 0.02^b^	63.26 ± 0.02^a^	67.74 ± 0.03^a^	7.89 ± 0.02^d^
PPI30-RS	59.81 ± 0.01^c^	63.15 ± 0.03^b^	67.23 ± 0.01^d^	7.87 ± 0.03^d^
PPI40-RS	59.83 ± 0.01^c^	63.14 ± 0.02^b^	67.41 ± 0.02^c^	7.91 ± 0.02^d^

Values with different letters in the same column are considered significant different (*p* < 0.05). T_o_, onset temperature; T_p_, peak temperature; T_c_, conclusion temperature; Δ*H*, gelatinization enthalpy; Abbreviations (RS, PPI, PPI0-RS, PPI10-RS, PPI20-RS, PPI30-RS, and PPI40-RS) are the same as those shown in Fig. 4.

The decrease in the Δ*H* value of RS after PPI addition can be explained by the three underlying mechanisms. First, PPI addition exerts a dilution effect, lowering the relative starch concentration in PPI–RS mixtures and decreasing the overall Δ*H* value. Second, water competition between PPI and RS affects water migration during gelatinization, weakening starch-water interactions and ultimately decreasing the Δ*H* value (Zhu et al., 2024). Third, PPI addition decreases the starch short-range molecular order (Fig. 4, Table 1), thereby reducing the energy required for gelatinization. As shown in Table 3, the PPI10-RS and PPI20-RS groups showed lower Δ*H* values than the PPI0-RS group. Ball-milled PPI with smaller particle size had higher abilities to compete water with starch and destroy the starch short-range molecular order, thus leading to a decrease in the starch Δ*H* value. However, when the milling time exceeded 20 min, the changes in Δ*H* value were no longer significant (*p* > 0.05). This lack of significance is likely attributed to the fact that PPI particle size no longer decreased markedly after 20 min ball milling, compared with the changes within the first 20 min.

### 3.5 Pasting properties

The pasting properties of the pure RS and PPI-RS mixtures are shown in Table 4. Compared with pure RS, addition of native PPI (PPI0) significantly (*p* < 0.05) decreased the PV, TV, FV, BD, and SB values. Further decreases were also observed in the PV, TV, FV, BD, and SB values when native PPI in PPI-RS mixtures was replaced by ball-milled PPI. Similar to our results, Liu, Liu, et al. (2021) found the addition of whey protein greatly reduced the PV, TV, FV, and BD values of potato starch. Sun and Xiong (2014) demonstrated that increasing the proportion of peanut protein isolate in pea starch-peanut protein blends induced reductions in key pasting parameters, including PV, FV, BD, and SB.

**Table 4:** Pasting properties of RS or PPI-RS mixtures.

Samples	PV/cp	TV/cp	BD/cp	FV/cp	SB/cp
RS	3848.3 ± 17.2^a^	2388.0 ± 5.6^a^	1460.3 ± 11.7^a^	4209.7 ± 9.0^a^	1726.0 ± 6.6^a^
PPI0-RS	3437.3 ± 16.6^b^	1991.0 ± 5.0^b^	1446.3 ± 13.6^b^	3525.7 ± 9.6^b^	1603.0 ± 6.3^b^
PPI10-RS	3402.0 ± 4.6^c^	1972.0 ± 1.0^c^	1430.0 ± 5.6^c^	3511.0 ± 6.1^b^	1560.0 ± 7.0^c^
PPI20-RS	3324.7 ± 9.5^e^	1901.3 ± 6.7^f^	1423.3 ± 16.5^cd^	3453.3 ± 7.8^c^	1507.0 ± 10.0^d^
PPI30-RS	3345.0 ± 10.8^d^	1944.0 ± 2.1^d^	1401.0 ± 9.6^d^	3452.7 ± 7.1^c^	1508.0 ± 6.7^d^
PPI40-RS	3346.7 ± 9.6^d^	1919.0 ± 1.7^e^	1427.7 ± 11.0^c^	3438.0 ± 3.6^c^	1517.0 ± 4.5^d^

Values with different letters in the same column are considered significant different (*p* < 0.05). Abbreviations (RS, PPI, PPI0-RS, PPI10-RS, PPI20-RS, PPI30-RS, and PPI40-RS) are the same as those shown in Fig. 4. PV, TV, FV, BD, and SB represent peak viscosity, trough viscosity, final viscosity, breakdown, and setback, respectively.

PV represents the maximum viscosity of starch paste at 95 °C, which reflects the water absorption and swelling capacity of starch granules during the heating process (Hedayati et al., 2020). The lower PV of RS after the addition of native or ball-milled PPI can be attributed to the dilution effect and water competition between PPI and starch. This competition limits starch granule swelling and amylose leaching, reduces friction between swollen granules, ultimately lowering the PV of starch paste (Gui et al., 2022). Moreover, ball-milled PPI-RS mixtures exhibited lower PV values than the native PPI-RS mixtures, indicating that ball-milling enhanced the ability of PPI to inhibit the swelling and gelatinization of starch.

BD is defined as the viscosity difference between PV and TV, which reflects the high-temperature shear resistance of starch paste. Ribotta et al. (2007) observed a positive correlation between BD and PV; both parameters are linked to granule swelling under heating. In this study, the reduced BD values after PPI addition indicates greater structural stability of starch granules. PPI wrapped around the surface of starch granules and played a protective role in the process of hydrothermal treatment (Lin et al., 2022).

SB is the viscosity recovery of starch and reflects the degree of starch retrogradation during the cooling stage. Incorporation of PPI significantly reduced the SB value of RS (*p* < 0.05), suggesting that the protein suppressed RS retrogradation. Hydrogen bonding, hydrophobic interactions, and electrostatic interactions between amylose and PPI disrupt amylose–amylose associations, thereby hindering amylose rearrangement and retrogradation (Chen et al., 2019). Compared with native PPI, ball-milling treatment of PPI further decreased the SB values of PPI-RS mixtures. This might be attributed to that ball-milling treatment modified the spatial structure and decreased the particle size of PPI, which strengthened PPI–RS interactions and inhibited starch retrogradation.

### 3.6 Textural properties of gel

The textural properties of pure RS and PPI-RS composite gels are presented in Table 5. Among all the gel samples, the pure RS gel showed the highest hardness, while the incorporation of PPI significantly reduced the gel hardness (*p* < 0.05). Such composite gels with lowered hardness have potential applications as nutritious, easy-to-swallow foods for individuals with dysphagia (Min et al., 2026). The hardness of starch gels is strongly dependent on amylose content, as higher degrees of cross-linking and entanglement between amylose molecules result in greater gel hardness (Gaines et al., 2000). Adding PPI decreased the relative amylose content in the gel system, thereby reducing gel hardness. Furthermore, PPI could bind with RS *via* hydrogen bonding, which suppressed the molecular rearrangement and retrogradation of RS and further reduced the hardness of composite gels (Luo et al., 2025). Compared with native PPI, ball-milled PPI induced a more significant reduction in the hardness of PPI-RS composite gels (*p* < 0.05). Ball-milling reduced the particle size of PPI, enhancing its binding capacity with starch granules. The enhanced intermolecular interactions between PPI and RS hindered the aggregation and rearrangement of starch molecules and weakened the intermolecular forces among amylose chains, ultimately lowering the hardness of composite gels. Ji et al. (2024) also reported that ultrasonically modified rice protein with smaller particle size and higher solubility could effectively reduce the hardness of rice protein-rice starch composite gels.

**Table 5:** Textural properties of RS or PPI-RS mixture gel.

Samples	Hardness (N)	Springiness	Cohesiveness	Gumminess (N)
RS	4.95 ± 0.06^a^	0.99 ± 0.01^a^	0.52 ± 0.01^a^	2.57 ± 0.03^a^
PPI0-RS	4.36 ± 0.04^b^	0.98 ± 0.01^a^	0.51 ± 0.01^a^	2.22 ± 0.02^b^
PPI10-RS	4.15 ± 0.08^c^	0.98 ± 0.01^a^	0.51 ± 0.01^a^	2.12 ± 0.04^c^
PPI20-RS	3.98 ± 0.06^d^	0.97 ± 0.01^a^	0.50 ± 0.01^a^	1.99 ± 0.02^d^
PPI30-RS	3.92 ± 0.07^d^	0.98 ± 0.01^a^	0.51 ± 0.01^a^	2.00 ± 0.03^d^
PPI40-RS	3.97 ± 0.05^d^	0.98 ± 0.01^a^	0.50 ± 0.01^a^	1.99 ± 0.02^d^

Values with different letters in the same column are considered significant different (*p* < 0.05). Abbreviations (RS, PPI, PPI0-RS, PPI10-RS, PPI20-RS, PPI30-RS, and PPI40-RS) are the same as those shown in Fig. 4.

Gumminess, a critical textural attribute of semi-solid foods, is defined as hardness × cohesiveness (Sun & Xiong, 2014). As Table 5 shown, the addition of PPI significantly decreased the gumminess of pure RS gels (*p* < 0.05), and ball-milling pretreatment of PPI further lowered the gumminess of the resultant PPI-RS composite gels. Springiness refers to the recovery rate of a material to its initial state after the removal of applied deformation force, whereas cohesiveness reflects the internal bonding strength of the sample matrix (Kadival et al., 2023). Sun and Xiong (2014) found that incorporating peanut protein isolate at concentrations below 20% exerted no significant effects (*p* > 0.05) on the springiness and cohesiveness of pea starch gels. In the present study, the addition level of both native PPI and ball-milled PPI was fixed at 10%. Consistent with the above observation, Table 5 shows that the incorporation of either native or ball-milled PPI had no significant effect (*p* > 0.05) on the springiness or cohesiveness of RS gels.

### 3.7 *In vitro* starch digestion

Numerous studies have demonstrated that the incorporation of exogenous proteins into starchy foods can reduce starch digestibility, which is beneficial for dietary intervention in diabetic patients. The *in vitro* digestibility of the pure RS and PPI-RS mixtures is presented in Table 6. The pure RS contained 69.51% of RDS, 16.32% of SDS, and 14.17% of resistant starch. After the addition of native PPI, the RDS content decreased to 66.22%, whereas the SDS and resistant starch contents increased to 17.73% and 16.04%, respectively. The incorporation of PPI significantly decreased the RS digestibility, which could be attributed to the following three mechanisms. First, proteins can bind to starch through non-covalent bonds, including hydrophobic interactions, hydrogen bonding, and electrostatic attraction. This binding enables the protein to act as a physical barrier, obstructing the interaction between starch molecules and digestive enzymes, thereby inhibiting starch hydrolysis (López-Barón et al., 2017). Second, PPI restrains the swelling and gelatinization of RS during hydrothermal processing, forming a denser physical barrier against digestive enzymes and further reducing starch digestibility. Third, PPI can bind to amylase in a non-catalytic manner, competitively inhibiting enzyme activity and suppressing the enzymatic hydrolysis of starch. Compared with the native PPI-RS group (PPI0-RS), 10 min and 20 min ball-milling treatments further decreased the RDS content while increasing the SDS and resistant starch contents of the composite mixtures. However, prolonging ball-milling beyond 20 min produced only minor, non-significant changes in RDS and SDS contents (*p* > 0.05). These results indicate that 20 min might be the optimal ball-milling duration to modify the digestive characteristics of PPI-RS composites.

**Table 6:** *In vitro* digestion of RS or RS in PPI-RS mixtures (%).

Samples	RDS	SDS	Resistant starch
RS	69.51 ± 0.08^a^	16.32 ± 0.17^d^	14.17 ± 0.10^e^
PPI0-RS	66.22 ± 0.09^b^	17.73 ± 0.13^c^	16.04 ± 0.14^d^
PPI10-RS	63.08 ± 0.25^c^	19.15 ± 0.19^b^	17.76 ± 0.07^b^
PPI20-RS	60.44 ± 0.08^d^	21.47 ± 0.14^a^	18.09 ± 0.11^a^
PPI30-RS	60.66 ± 0.05^d^	21.49 ± 0.16^a^	17.85 ± 0.13^b^
PPI40-RS	60.99 ± 0.10^d^	21.46 ± 0.07^a^	17.54 ± 0.08^c^

Values with different letters in the same column are considered significant different (*p* < 0.05). RDS, rapidly digestible starch; SDS, slowly digestible starch; Abbreviations (RS, PPI, PPI0-RS, PPI10-RS, PPI20-RS, PPI30-RS, and PPI40-RS) are the same as those shown in Fig. 4.

This trend likely occurs because, during the first 20 min of milling, the PPI undergoes significant particle size reduction and a corresponding increase in specific surface area. This allows the protein to bind more to starch granules. Additionally, the exposure of internal hydrophobic groups strengthens the protein-starch interaction, further hindering amylase accessibility to starch (López-Barón et al., 2018). However, when milling time exceeds 20 min, particle size reduction becomes less pronounced. At this stage, intermolecular forces may cause the protein to reconfigure into new structures, weakening its interaction with starch compared with optimal state at 20 min.

## 4 Conclusion

Compared with native PPI, incorporation of ball-milled PPI lowered the short-range molecular order, swelling power and water absorption capacity of PPI–RS mixtures, while enhancing their solubility. DSC and RVA results demonstrated that ball-milling treatment of PPI suppressed the swelling and gelatinization degree of starch in PPI–RS composites under hydrothermal conditions. TPA tests showed that ball-milling treatment of PPI reduced the hardness of the composite gel. *In vitro* digestion assays indicated that ball-milling treatment of PPI decreased RDS and SDS, and increased resistant starch contents. No further significant changes (*p* > 0.05) in gelatinization enthalpy, setback, gel hardness and RDS were detected beyond 20 min of ball milling, revealing 20 min as the critical threshold for optimizing the physicochemical properties of PPI–RS blends. Overall, moderate ball-milling pretreatment of PPI effectively strengthens starch–protein intermolecular interactions, which can facilitate the development of high-nutrition, low-digestibility starch-based foods.

Regarding the limitations of this study, only ball-milling was employed to modify PPI and strengthen protein–starch interactions in the present work. Other modification approaches, including deep eutectic solvents, sonication, and homogenization, can also be applied to improve the physicochemical properties of starch–protein composites in future studies. Furthermore, protein–starch complexes exhibit promising potential applications for food industry, such as easy-to-swallow foods for individuals with dysphagia, low-glycemic foods, and matrices for encapsulating bioactive components, which need further investigated in the future studies.

## CRediT authorship contribution statement

**Jun Jiang:** Writing – original draft, Visualization. **Yirong Zhang:** Methodology, Investigation. **Jun Wang:** Methodology, Investigation. **Derui Kong:** Methodology, Investigation. **Tianyu Yang:** Writing – review & editing. **Zuohua Zhu:** Writing – review & editing, Funding acquisition. **Naifu Wang:** Writing – review & editing.

## Declaration of competing interest

The authors declare that they have no known competing financial interests or personal relationships that could have appeared to influence the work reported in this paper.

## Data Availability

Data will be made available on request.
